# Supplementary material for: Hyperthermia Increases Neurotoxicity Associated with Novel Methcathinones
Source: Cells. 2020 Apr 14;9(4):965. doi: 10.3390/cells9040965 (PMC7227000; doi:10.3390/cells9040965)
Supplement: Supplementary file 1 [file cells-09-00965-s001.pdf]

| MC | **4-FMC** | **4-CMC** | **4-MMC** |
|---|---|---|---|
| 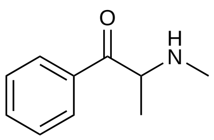 | 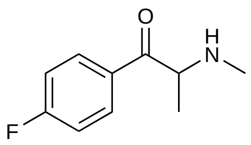 | 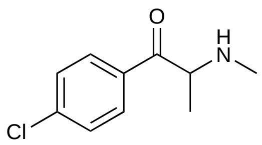 | 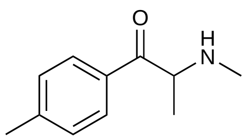 |
| **Amphetamine** | **4-FA** | **PCA** | **MDMA** |
| 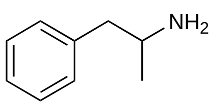 | 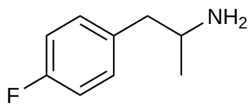 | 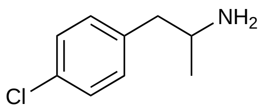 | 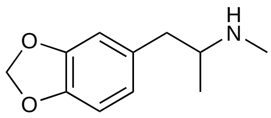 |

**Figure S1.** Chemical structures of amphetamine and methcathinone derivatives.

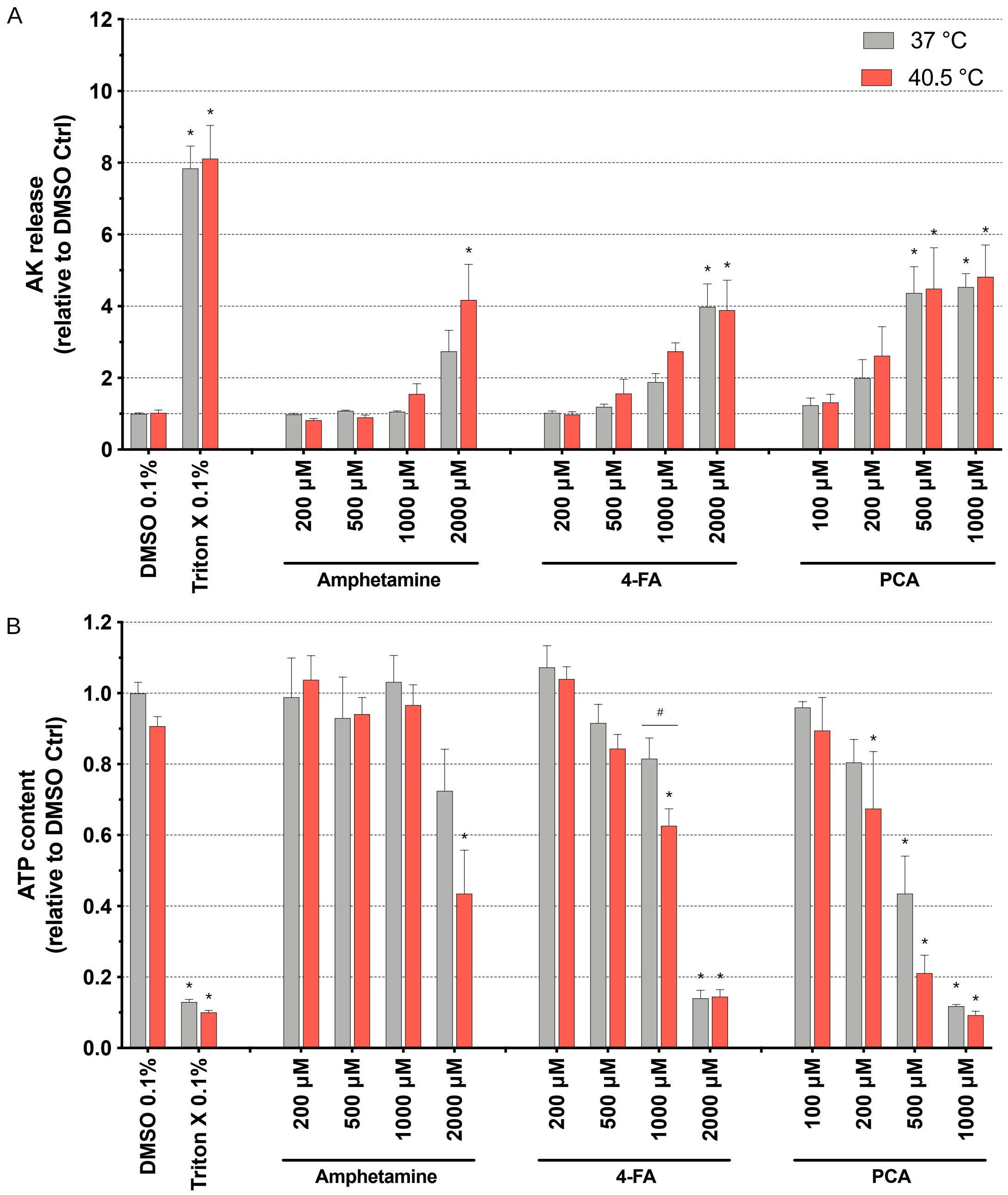


**Figure S2.** (**A**) Plasma membrane integrity and (**B**) intracellular ATP content assessed in SH-SY5Y cells after 24 h of exposure at 37 °C and 40.5 °C to amphetamine, 4-FA (200–2000 µM), and PCA (100–1000 µM). DMSO and Triton X were used as negative and positive controls, respectively. Data are expressed relative to DMSO control incubations as mean ± SEM of eight independent experiments run in quadruplicate. Statistical comparisons were performed with one-way ANOVA followed by *t*-tests (**P* ≤ 0.05 *versus* control at the same temperature; #*P* ≤ 0.05 *versus* the same concentration at different temperature).

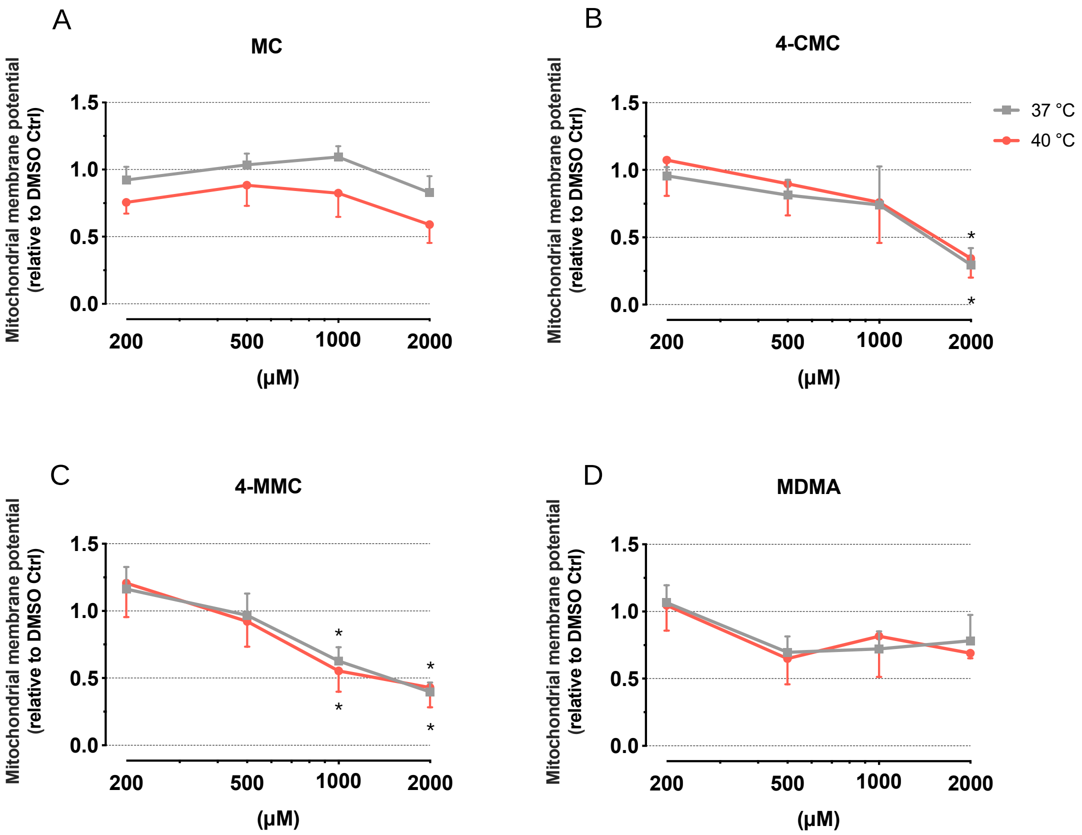


**Figure S3.** Mitochondrial membrane potential in SH-SY5Y cells. The mitochondrial membrane potential was measured after 24 h of exposure at 37 °C or 40.5 °C to MC, 4-CMC, 4-MMC, and MDMA (200–2000 µM). Data are expressed relative to DMSO control cells as mean ± SEM of three independent experiments run in quadruplicate. Statistical comparisons were performed with one-way ANOVA followed by *t*-test (**P* ≤ 0.05 *versus* control at the same temperature).

.

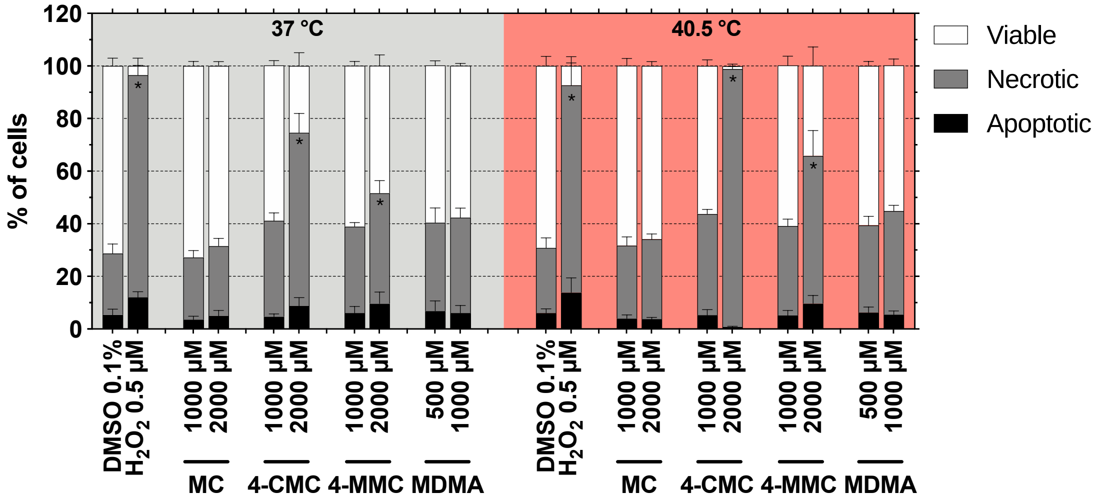


**Figure S4.** Percentage of viable, necrotic and apoptotic cells after 24 h of exposure to test compounds. SH-SY5Y cells were exposed to MC, 4-CMC, 4-MMC (1000 μM and 2000 μM), and MDMA (500 μM and 1000 μM) for 24 h at 37 °C or 40.5 °C. DMSO and $H_2O_2$ were used as negative and positive controls, respectively. Data are expressed as mean ± SEM of six independent experiments. Statistical comparisons were performed with one-way ANOVA followed by *t*-tests (**P* ≤0.05 *versus* control at the same temperature).

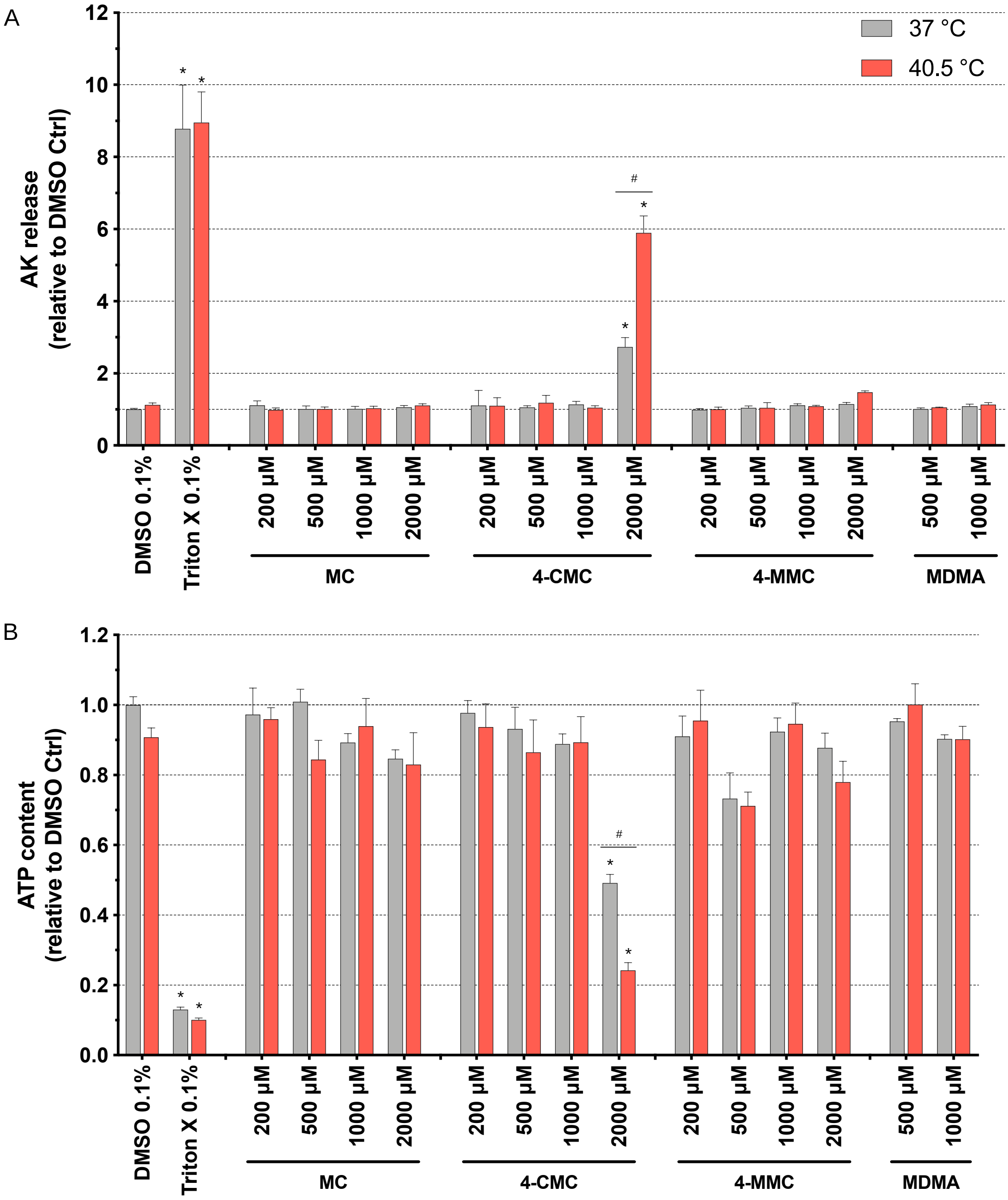


**Figure S5.** (**A**) Plasma membrane integrity and (**B**) intracellular ATP content assessed in SH-SY5Y cells after 6 h of exposure at 37 °C and 40.5  C to MC, 4-CMC, 4-MMC (200–2000 µM), and MDMA (500 µM and 1000 µM). DMSO and Triton X were used as negative and positive controls, respectively. Data are expressed relative to DMSO control incubations as mean ± SEM of eight independent experiments run in quadruplicate. Statistical comparisons were performed with one-way ANOVA followed by *t*-tests (*$P$ ≤0.05 *versus* control at the same temperature; #$P$ ≤0.05 *versus* the same concentration at different temperature).

**Table S1.** Quantification ($IC_{50}$) of membrane toxicity (MT) and ATP depletion (ATP) by methcathinones at 37 °C and 40.5 °C in SH-SY5Y cells.

| | **37°C** | | | **40.5°C** | | |
|---|---|---|---|---|---|---|
| | MC (mM) | 4-CMC (mM) | 4-MMC (mM) | MC (mM) | 4-CMC (mM) | 4-MMC (mM) |
| MT $IC_{50}$ | >2 | >2 | >2 | >2 | 1.94 | 1.57 |
| ATP $IC_{50}$ | >2 | 1.30 | >2 | >2 | 0.77 | 0.86 |
